# Editorial

**Published:** 2019-02-01

**Authors:** Prof. Hasan ÖzKAN

**Affiliations:** President of Euroasian Gastroenterological Association Editor in Chief of Euroasian Journal of Hepato-Gastroenterology Head of Hepato-Gastroenterology Department Ankara University School of Medicine, Turkey

Distinguished colleagues

We founded the Euroasian Gastroenterological Association 23 years ago in Ankara. Our aim has been to introduce scientists and the science of Central Asian countries with the West, to form a Euroasia science bridge and create a new scientific platform, in other words a new international scientific association. The first event of this new scientific vision took place on May 21-25, 1997 in Baku, the capital of Azerbaijan. We brought together many scientists from Turkey, Azerbaijan, Russia, Uzbekistan, Kazakhstan, Kyrgyzstan, Turkmenistan and Georgia. When we look back after all those years, we are happy to see the importance of our initiative in 1997.

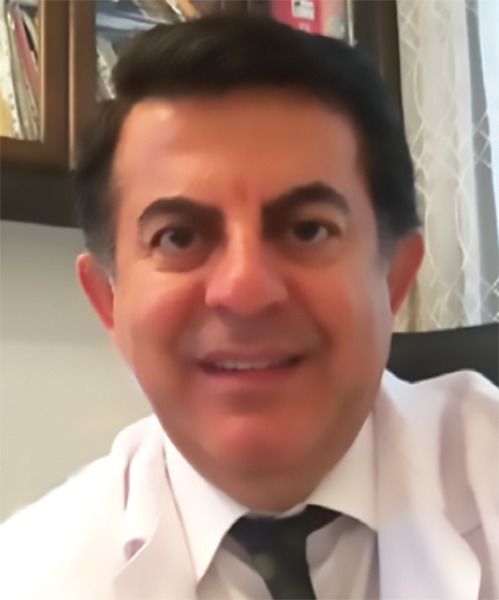



That was just a dream 23 years ago. Today we see that it has come through. We decided to organise international scientific congresses with a view to achieve our goals and objectives. We have organized 16 international congresses, three single topic conferences and three international conferences so far. These are as follows:

1. 1997 Baku-Azerbaijan, 2. 1998 Almati-Kazakhstan, 3. 1999 Antalya-Turkey, 4. 2000 Tashkent-Uzbekistan, 5. 2001 Bishkek-Krygyzhstan, 6. 2003 Baku-Azerbaijan, 7. 2004 Ankara-Turkey, 8. 2005 Tbilisi-Georgia, 9. 2006 Baku-Azerbaijan,10. 2007 Isfahan-Iran, 11. 2008 Baku-Azerbaijan, 12. 2011 Baku-Azerbaijan, I. STC 2013 Yalta, Crimea, 13. 2013 Baku-Azerbaijan, II. STC 2014 Ljubljana-Slovenia, 14. 2014 Magusa-Nothern Cyprus, III. STC 2015 Bucharest - Romania, 15. 2016 Antalya-Turkey, 1st Conference of EGA 2016, 16. 2017 Mostar-Bosnia Herzegovina, 2nd Conference of EGA 2017 Almaty - Kazakhstan, 3rd Conferance of EGA 2017 Antalya-Turkey.

We are going to organize two Euroasian congresses this year. These will be held in Split-Croatia (17th) and Baku-Azerbaijan (18th).

We have achieved the impossible with scarce resources. We will see the importance of these congresses clearly in the next years. We believe that our young colleagues will take this further.

A few years ago we decided to publish a journal in order to achieve our objectives. To this end Dr. Sheikh Mohammad Fazle Akbar from Japan, Prof. Mamun Al Mahtab from Bangladesh and I began to publish Euroasian Journal of Hepato-Gastroenterology with Jaypee Brothers Medical Publishers (P) Ltd. (New Delhi, India). Numerous articles have been published among papers submitted by scientists from 37 different countries in the world. We applied for the well-known American index, PubMed and finally entered PubMed and PMC in December 2017. This was yet-another achievement for our association. Lately, we applied for International Scientific Index (ISI) at the end of 2018. I believe that the application will be accepted shortly.

We organized a very fruitful congress in Mostar-Bosnia Herzegovina last year with the help of Prof. Enver Zerem and Prof. Milenko Bevanda. This year we are going to organize the 17th Euroasian Congress of Hepato-Gastroenterology and Surgery at Split, Crotia on March 27-31 with the kind support of Prof. Neven Ljubicic, Prof. Miroslav Smunic and many other Croatian scientists.

This year also the 18th Euroasian Congress of Hepato-Gastroenterology and Surgery (Azerbaijan) will take place in the beginning of October at Baku in the memory of Prof. Büyükkişi Agayev, one of the pionners of our Euroasian vision. I would like to thank our respected Azerbaijani colleagues in advance, including Prof. Geray Geraybeyli, the rector of the Azerbaijan University of Medicine and Prof. Rauf Agayev.

I will be expecting your support and contributions in taking our scientific vision further and offer my kind regards to all of my colleagues in Euroasia.

**Prof. Hasan ÖzKAN**

President of Euroasian Gastroenterological Association

Editor in Chief of Euroasian Journal of Hepato-Gastroenterology

Head of Hepato-Gastroenterology Department

Ankara University School of Medicine, Turkey

